# Chemerin Is Induced in Non-Alcoholic Fatty Liver Disease and Hepatitis B-Related Hepatocellular Carcinoma

**DOI:** 10.3390/cancers12102967

**Published:** 2020-10-13

**Authors:** Elisabeth M. Haberl, Susanne Feder, Rebekka Pohl, Lisa Rein-Fischboeck, Kerstin Dürholz, Laura Eichelberger, Josef Wanninger, Thomas S. Weiss, Christa Buechler

**Affiliations:** 1 Department of Internal Medicine I, Regensburg University Hospital, 93053 Regensburg, Germany; Haberl.elisabeth@gmx.de (E.M.H.); Feder.susanne@gmx.de (S.F.); becky-pohl@web.de (R.P.); lisa.rein-fischboeck@gmx.de (L.R.-F.); kerstin.duerholz@uk-erlangen.de (K.D.); laura.eichelberger@helmholtz-muenchen.de (L.E.); josef.wanninger@gmx.net (J.W.); 2 Children’s University Hospital (KUNO), Regensburg University Hospital, 93053 Regensburg, Germany; thomas.weiss@klinik.uni-regensburg.de

**Keywords:** CMKLR1, hepatitis, tazarotene, chemerin isoform

## Abstract

**Simple Summary:**

Hepatocellular carcinoma (HCC) is a frequent liver cancer and high expression of bioactive chemerin in hepatocytes was protective in experimental HCC models. The main risk factors for HCC are non-alcoholic fatty liver disease (NAFLD), hepatitis B and C infections. The current analysis showed that chemerin protein was induced in HCC tissues of NAFLD and hepatitis B infected patients. This upregulation was modest in patients with unknown disease etiology and not detected in hepatitis C infected patients. Protein levels of the chemerin receptor CMKLR1 strongly declined in the tumors of NAFLD patients and patients with unclear disease etiology but not in patients with viral infections. Our results demonstrate that the expression of chemerin in HCC is related to disease etiology and this could also apply to the role of chemerin in human HCC. In contrast to the present findings, chemerin was shown to be low in the HCC tissues of Asian patients with mostly viral disease etiology and this indicates ancestry-specific regulation of chemerin in HCC.

**Abstract:**

Chemerin is protective in experimental models of hepatocellular carcinoma (HCC). Noteworthy, chemerin mRNA and protein were reduced in HCC tissues of Asian patients with mostly hepatitis B disease etiology. The current study nevertheless showed that chemerin protein was induced in tumor tissues of European HCC patients with non-alcoholic fatty liver disease (NAFLD) and patients with unclear disease etiology. A similar regulation was observed in hepatitis B virus (HBV), but not in hepatitis C virus (HCV), related HCC. The apparent discrepancy between the regulation of chemerin in HBV-HCC obtained from our study and recent reports led us to use the chemerin antibodies applied in the previous assays. These antibodies could not equally detect different chemerin isoforms, which were overexpressed in HepG2 cells. Higher chemerin protein in HCC was nevertheless confirmed by the use of all antibodies. Chemerin protein was low in Huh7 and PLC/PRF/5 cells whereas HepG2 and Hep3B cells had chemerin protein similar as primary human hepatocytes. Besides, the anti-tumor effects of retinoids in hepatocyte cell lines did not enclose upregulation of chemerin, which was initially discovered as a tazarotene induced protein in the skin. Finally, protein levels of the chemerin receptor, chemokine-like receptor 1 (CMKLR1), declined in non-viral, and tended to be lower in HBV-HCC tissues suggesting reduced chemerin activity in the tumors. To sum up, our work showed an opposite regulation of chemerin and CMKLR1 in NAFLD and HBV associated HCC. In HCV-HCC neither chemerin nor its receptor were changed in the tumor tissues. Current findings do not support a critical role of total chemerin protein levels in HCC of non-viral and viral etiology. Accordingly, tumor-localized chemerin protein was not associated with tumor-node-metastasis classification.

## 1. Introduction

The chemoattractant chemerin is highly expressed in adipocytes and hepatocytes [1]. Chemerin serum levels are increased in obesity, consistent with its expression in adipose tissues. Chemerin is a multifunctional adipokine and regulates insulin sensitivity of skeletal muscles, hepatic gluconeogenesis, adipogenesis, cell proliferation and angiogenesis [1]. Chemerin has pro- and anti-inflammatory activities, depending on the model studied [1,2,3]. It also has a role in carcinogenesis, and again, pro- as well as anti-tumorigenic functions were identified [4,5]. Chemerin is chemotactic for different cells, and controls the migration of immune cells. Recruitment of natural killer cells by chemerin suppressed melanoma [6]. In experimental models of hepatocellular carcinoma (HCC) the biologically highly active murine chemerin isoform–chemerin-156–protected from tumor growth and metastasis [7,8,9]. The tumor-inhibitory effect of chemerin in HCC was reduced in Rag1^-/-^ animals indicating that T cells were involved [9].

It is well known that cells secrete inactive prochemerin, which has to be activated by C-terminal processing. Different proteases produce bioactive chemerin variants and further processing to shorter isoforms by some of these enzymes contributes to chemerin inactivation [1]. Analysis of chemerin protein levels by commercially available antibodies can not distinguish these different chemerin isoforms. Increased serum chemerin in obesity did not match the ex-vivo measured activation of chemokine-like receptor 1 (CMKLR1) [10]. In fact, degraded chemerin appeared in the obese [11] showing that measurement of total protein levels does not specify chemerins’ activity. Given that the majority of serum chemerin exists as prochemerin it is reasonable to speculate that chemerin activation is initiated at the sites of injury [10,11]. Recent research suggests that chemerin secreted by tumor cells or the tumor surrounding tissues exerts autocrine and / or paracrine effects [4].

Experiments on chemerin expression in hepatocellular carcinoma (HCC) were conducted in 2011 by a group from China. This analysis showed a decline of chemerin protein in cancer tissues in about 60% of the patients. No significant differences were found in the tissues of the remaining patients [12]. This analysis enrolled 124 patients and 103 of them had a positive hepatitis history. Chemerin protein expression in the tumors positively correlated with infiltration of dendritic and natural killer cells. Disease free survival was significantly better in patients with high tumor chemerin [12]. Of the 159 Chinese HCC patients analyzed in a separate study, 139 were infected with hepatitis B virus (HBV) and one patient with hepatitis C virus (HCV) [9]. In this HCC cohort, chemerin protein was again low in the tumors. Chemerin protein expression was positively associated with low-grade tumor differentiation, small tumor size and early Barcelona Clinic Liver Cancer stage. Importantly, patients with high chemerin in the tumors had better survival [9].

A third study from China confirmed that chemerin was suppressed in liver cancer. Again, patients with higher tumor chemerin protein had better survival [8]. Of the study population 197 patients were hepatitis B positive. Disease etiology of the remaining 93 patients was not related to HBV infections [8]. Patients without viral infection and patients without liver cirrhosis more often had higher chemerin expression. More importantly, tumor chemerin protein was not related to tumor size or tumor-node-metastasis (TNM) classification in this cohort [8].

Analysis of liver cancer cell lines supported the negative association of chemerin protein with HCC. The cell lines Huh7, HCC-97L and HCCLM3 had very little soluble chemerin protein in comparison to LO2 cells, which is a fetal hepatocyte cell line derived from healthy liver [9]. Moreover, immunoblot analysis of various tumor cells detected chemerin in HepG2 cells but no further tumor cell [8].

All of these tumor cells expressed CMKLR1 [8]. CMKLR1 is one of the chemerin receptors described so far. G-protein-coupled receptor 1 (GPR1) is a less well studied chemerin receptor [1,4]. Expression of CMKLR1 and GPR1 in liver cancers was not analyzed in detail so far.

Large geographic variations exist in HCC disease etiology. In Asia HBV is endemic and about 77% of liver cancers are caused by chronic HBV infection, 3% are related to HCV and 1% to non-alcoholic steatohepatitis (NASH) [13]. In Germany about 20% of HCCs are associated with HCV, 25% with HBV and 22% with NASH [14].

Non-alcoholic fatty liver disease (NAFLD) is a risk factor for HCC. NASH depicts a more severe form of this disease and may progress to liver cirrhosis. Of note, HCC develops in the cirrhotic and the non-cirrhotic NASH liver [15,16]. Prevalence of NAFLD is about 25% in Western countries and Asia. NASH is a relevant cause of HCC in the Western world with an absolute risk of 1–4% per year. In Asia the absolute risk of NASH-HCC is < 0.1% per year and viral cirrhosis remains the main etiology in Asia [15,17]. The number of obese in Asian countries rapidly increased during the last two decades [17]. The low prevalence of NASH-HCC in Asia may be related to the later onset of the obesity pandemic and thus a shorter disease duration in Asian than Western countries [17].

Previous studies described increased and decreased chemerin mRNA expression in the human NASH liver [18,19,20]. In fact, regulation of hepatic chemerin protein in human NASH is still a matter of debate [18]. Very little is known about chemerin in NASH-HCC [21]. In an experimental NASH-HCC animal model chemerin protein levels were comparable in paratumor and tumor tissues [21]. In the present study, chemerin and CMKLR1 protein levels were analyzed in NAFLD-HCC and for comparison in cryptic, HBV and HCV patients with HCC.

## 2. Results

### 2.1. Chemerin in Primary Human Hepatocytes and Hepatocyte Cell Lines.

**Figure 1:**
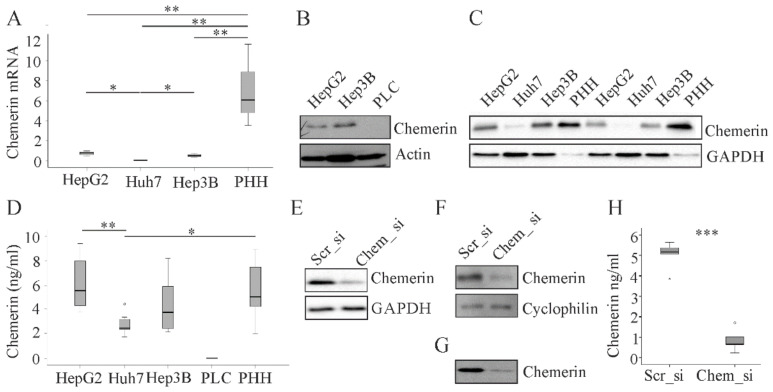
Chemerin in primary human hepatocytes (PHH) and hepatocyte cell lines. (**A**) Chemerin mRNA in HepG2, Huh7 and Hep3B cells and PHH (n = 3–6); (**B**) and (**C**) Chemerin protein in HepG2, Hep3B, PLC/PRF/5 (PLC), and Huh7 cells and PHH; (**D**) Chemerin in supernatants of HepG2, Huh7, Hep3B and PLC/PRF/5 (PLC) cells and PHH (n = 7–8); (**E**) Chemerin in HepG2 cells transfected with scrambled (scr) or chemerin (chem) siRNA; (**F**) Chemerin in PHH transfected with scrambled or chemerin siRNA; (**G**) Chemerin in the supernatant of PHH transfected with scrambled or chemerin siRNA; (**H**) Chemerin in the supernatant of PHH transfected with scrambled or chemerin siRNA measured by ELISA (n = 6 different donors). * *p* < 0.05, ** *p* < 0.01, *** *p* < 0.001. Statistical test used: unpaired and paired Student´s t-test.

In line with low levels of chemerin in human HCC tissues recent studies showed absent or little expression of chemerin in human HCC cell lines including Huh7 and Hep3B cells. The main limitation of these studies was that primary human hepatocytes (PHH) were not analyzed in parallel [8,9]. Here we show that chemerin mRNA was reduced in HepG2 (*p* < 0.01), Huh7 (*p* < 0.01) and Hep3B (*p* < 0.01) cells when compared to PHH (Figure 1A). 

Chemerin mRNA was least abundant in Huh7 cells (*p* < 0.01 when compared to PHH and *p* < 0.05 when compared to HepG2 and Hep3B cells) (Figure 1A). Immunoblot analysis could not detect chemerin protein in lysates of PLC/PRF/5 cells (Figure 1B). Chemerin protein expression was rather low in Huh7 cells (Figure 1C) but did not show gross differences between HepG2 cells, Hep3B cells and PHH (Figure 1B,C). Measurement of chemerin in cell supernatants by ELISA gave comparable results: Chemerin was not detected in supernatants of PLC/PRF/5 cells, and soluble protein was lower in Huh7 cells than PHH (*p* < 0.05) and HepG2 cells (*p* < 0.01). Hep3B and HepG2 cells essentially had the same concentration of soluble chemerin protein as the primary cells (Figure 1D).

These unexpected results lead us to review the specificities of the assays used to measure chemerin. One approach was the knock-down of chemerin with siRNA in HepG2 cells and PHH. Cells transfected with the chemerin specific siRNA had lower chemerin protein in the cell lysates (Figure 1E,F). Supernatant of PHH as analyzed by immunoblot and ELISA (*p* < 0.001) had greatly reduced chemerin protein in chemerin siRNA transfected cells (Figure 1G,H). These experiments confirmed that the assays specifically detect chemerin protein.

### 2.2. Chemerin in Human Non-Viral HCC

Having validated the specificity of the chemerin antibody for immunoblot analysis we next determined chemerin protein in human HCC. Therefore, paired tumor and para-tumorous tissues of 21 male patients not infected with hepatitis B or C virus were analyzed (Table 1). Tissues were obtained during liver resection surgery. In this cohort six patients had fatty liver and four patients had NASH indicating metabolic-syndrome associated HCC. Disease etiology of the further patients remained unknown.

**Figure 2:**
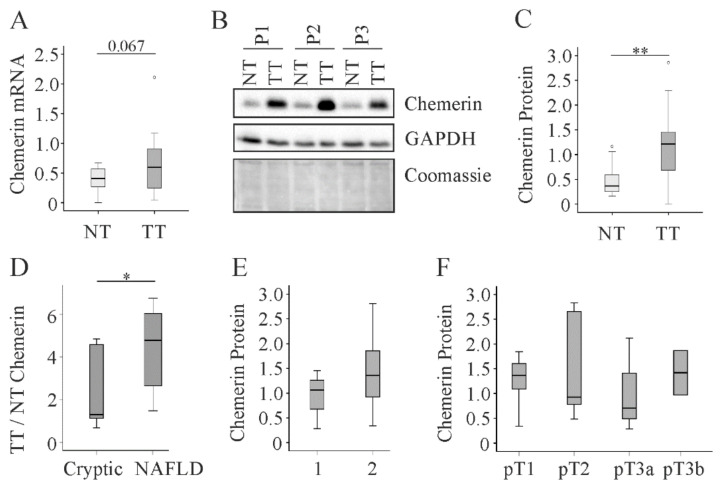
Chemerin in non-tumor (NT) and tumor tissue (TT) of 21 HCC patients with non-viral disease etiology. (**A**) Chemerin mRNA, the number in the figure indicates the p-value; (**B**) Chemerin protein in NT and TT of three different patients (P1 to P3); (**C**) Quantification of chemerin protein which was analyzed by immunoblot in 21 patients. GAPDH was used for normalization; (**D**) Ratio of TT to NT chemerin protein in patients with cryptic disease etiology (n = 11) or NAFLD (n = 10); (**E**) Chemerin in tumors of patients with grade 1 (well differentiated tumor) or grade 2 (moderately differentiated tumor); (**F**) Chemerin protein in the tumors of 20 patients stratified for TNM classification. * *p* < 0.05; ** *p* < 0.01. Statistical test used: Mann-Whitney U test and one-way ANOVA with posthoc Bonferroni.

Chemerin mRNA tended to be higher in the tumors and chemerin protein was induced in the cancer tissues (*p* < 0.01) (Figure 2A–C). The power value of this comparison was 0.99, which means a 99% probability of rejecting the null hypothesis. Moreover, ratio of tumor to non-tumorous chemerin was higher in patients with NAFLD (power value: 0.98 for comparison of non-tumor and tumor tissues, Table 2) than patients with cryptic disease etiology (*p* < 0.05; power value: 0.51 for comparison of non-tumor and tumor tissues, Table 2) (Figure 2D). The low power for cryptic HCC indicates that chemerin may not be induced in non-NAFLD HCC tissues and this has to be verified in large cohorts. Chemerin protein levels in HCC were not correlated with grading or TNM stage (Figure 2E,F).

### 2.3. Chemerin in Human Viral HCC

**Figure 3:**
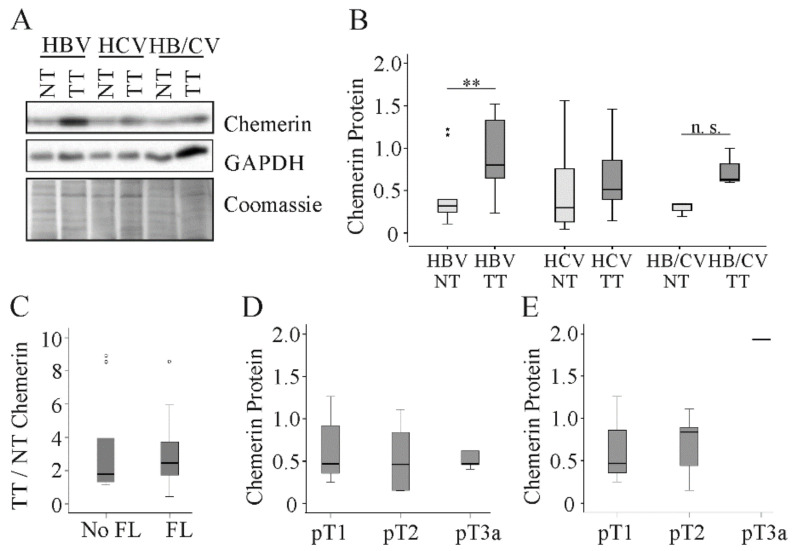
Chemerin in non-tumor (NT) and tumor tissue (TT) of 24 HCC patients with viral disease etiology. (**A**) Chemerin protein of three different patients. (**B**) Quantification of chemerin protein in 10 HBV, 11 HCV and 3 HBV/HCV infected patients. Coomassie stained membrane was used for normalization. (**C**) Ratio of TT to NT chemerin protein in 15 patients without and eight patients with fatty liver (FL). (**D**) Chemerin protein stratified for TNM classification in 23 patients. (**E**) Chemerin protein stratified for TNM classification in HBV infected patients (there was one patient with pT3a, and statistical test was not possible for this comparison). n.s. not significant. ** *p* < 0.01. Statistical test used: Mann-Whitney U test and one-way ANOVA with posthoc Bonferroni.

Previous studies reported decreased expression of chemerin in HCC tissues. The patients enrolled in these studies mostly had HBV-related etiology of HCC [8,9,12]. To clarify whether low tumor chemerin is a characteristic of HBV and possibly HCV related HCC, chemerin protein was determined in tissues of hepatitis virus infected patients (Table 1). In the ten patients with HBV chemerin protein was upregulated in HCC tissues (Figure 3A,B). The power value of this observation was 0.88, Table 2. In the 11 HCV-positive patients chemerin levels were essentially the same in tumor and para-tumorous tissues (Figure 3A,B and Table 2). In the three patients simultaneously infected with HBV and HCV, chemerin protein tended to increase in the tumors (Figure 3A,B and Table 2). In viral HCC, induction of chemerin protein in the tumor tissues was not altered with fatty liver, which is defined as steatosis in more than 5% of hepatocytes [22] (Figure 3C). Chemerin protein in the tumors was not related to grading or TNM classification in the whole cohort and when HCV positive patients were excluded (Figure 3D,E).

### 2.4. Detection of Chemerin Isoforms by Different Antibodies.

Chemerin is cleaved at its C-terminus to produce different isoforms, which may vary in their reactivity with the antibodies used for immunoblotting. R&D Systems offers a polyclonal goat and a monoclonal mouse antibody for immunoblot experiments with chemerin. For their chemerin detection, Lin *et al*. used the polyclonal goat antibody from R&D Systems [9]. This antibody was used to obtain the data shown in Figure 1, Figure 2 and Figure 3 of the current examinations. In their 2018 paper, Li et al. applied a not further disclosed antibody from R&D Systems [8]. In a third study a mouse polyclonal antibody from Abcam was employed [12]. Subsequently, we used the two antibodies from R&D Systems and the polyclonal antibody from Abcam to detect recombinant chemerin isoforms.

**Figure 4:**
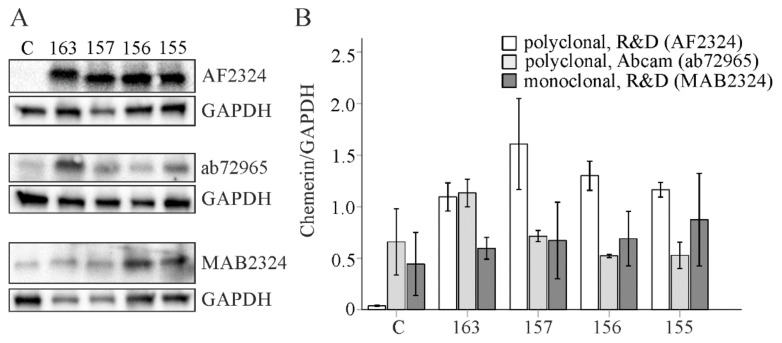
Analysis of chemerin variants by immunoblot. (**A**) Human chemerin isoforms 163, 157, 156 and 155 were overexpressed in HepG2 cells and protein was detected by three different antibodies (C, control-transfected cells). (**B**) Quantification of chemerin protein (n = 2).

Full-length human chemerin (163) and the processed isoforms (157,156,155) were expressed in HepG2 cells. The cell lysates were prepared, separated by SDS-Page and transferred to membranes, which were subsequently incubated with the antibodies. Upon short exposure time (1 second) of the membranes signals were detected when the polyclonal antibody from R&D Systems was used (this was shown recently by our group [23]. Longer exposure time (up to 400 seconds) was necessary to obtain signals with the other two antibodies (Figure 4A,B). At least with the use of our immunoblot protocol the polyclonal antibody from R&D Systems had a good signal-to-noise ratio. Importantly, this antibody detected all the different chemerin variants. The monoclonal antibody from R&D Systems and the polyclonal antibody from Abcam could not really confirm higher expression of all chemerin isoforms in the transfected cells (Figure 4A,B). The polyclonal antibody from Abcam was most reactive with prochemerin. The monoclonal antibody recognized above all the small chemerin isoforms. Thus, the polyclonal antibody from R&D Systems was most suited to detect chemerin by the immunoblot protocol used. Moreover, these experiments revealed that this chemerin antibody was appropriate to analyze different chemerin isoforms.

### 2.5. Detection of Chemerin in Human HCC Tissues by Different Antibodies

**Figure 5:**
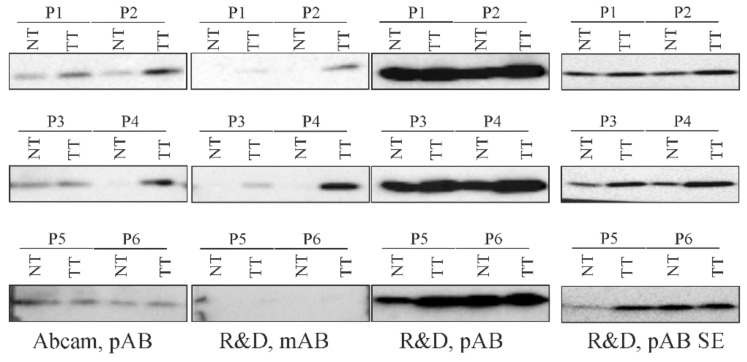
Chemerin protein in non-tumor (NT) and tumor tissue (TT) of HCC patients. Chemerin protein in NT and TT of six patients (P1 to P6) was analyzed with three different antibodies. pAB, polyclonal antibody; mAB monoclonal antibody; SE, short exposure time of the membrane.

Furthermore, we used these three antibodies to analyze human HCC and para-tumorous tissues. All antibodies identified increased chemerin protein in tumors of patient 1, 2, 3 and 4. The degree of chemerin induction in the tumors varied when identical tissues were analyzed by different antibodies (Figure 5).

Patients 5 and 6 had similar chemerin protein in HCC and non-tumorous tissues when analyzed with the antibody from Abcam and the polyclonal antibody from R&D Systems. Here, the monoclonal antibody from R&D Systems did not give any signal (Figure 5). In summary, this preliminary analysis showed that the three different antibodies could detect higher chemerin in human liver tumors.

### 2.6. CMKLR1 in HCC

**Figure 6:**
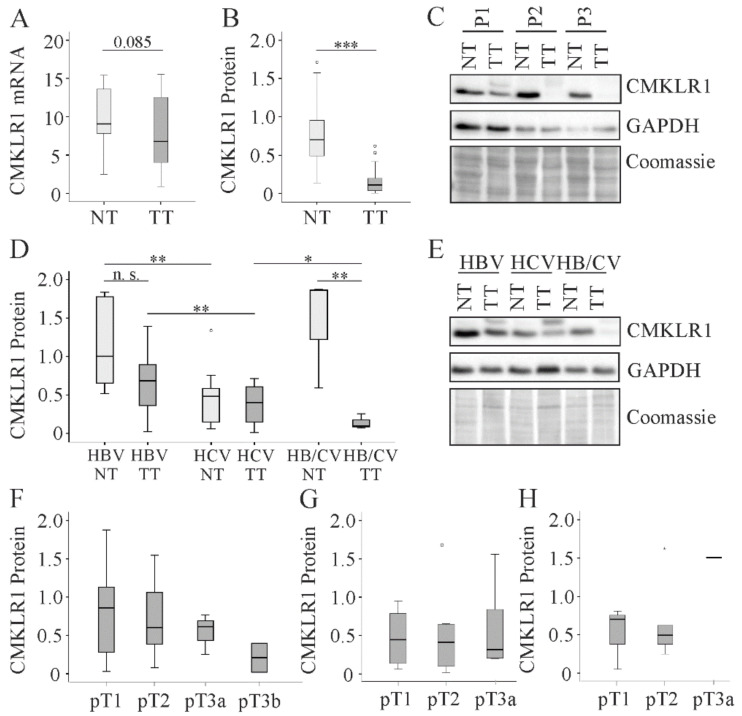
CMKLR1 in non-tumor (NT) and tumor tissue (TT) of HCC patients (**a**) CMKLR1 mRNA of 21 patients with non-viral disease etiology, the number in the figure depicts the p-value; (**b**) Quantification of CMKLR1 protein of 21 patients with non-viral disease etiology. Coomassie stained membrane was used for normalization; (**c**) CMKLR1 protein of three different patients (P1 to P3); (**d**) Quantification of CMKLR1 protein in NT and TT of 10 HBV, 11 HCV and three HBV/HCV infected patients. Coomassie stained membrane was used for normalization; (**e**) CMKLR1 protein of three different patients; (**f**) CMKLR1 protein stratified for TNM classification in patients with non-viral disease etiology. (**g**) CMKLR1 protein stratified for TNM classification in patients with viral disease etiology; (**h**) CMKLR1 protein stratified for TNM classification in HBV infected patients. N. s. not significant: * p < 0.05, ** p < 0.01, *** p < 0.001 Statistical test used: Mann-Whitney U test and one-way ANOVA with post-hoc Bonferroni.

CMKLR1 is the best studied chemerin receptor [24], but its expression has not been analyzed in human HCC tissues to our knowledge so far. CMKLR1 mRNA tended to be lower in tumor tissues of patients with non-viral HCC and protein expression was significantly reduced when compared to the respective para-tumorous tissues (Figure 6A–C). Power value for CMKLR1 protein change in HCC was 0.99. CMKLR1 protein was reduced in cryptic (from a median value of 0.7 to 0.03, p < 0.001, power = 0.96, Table 2) and NAFLD-HCC (from a median value of 0.6 to 0.1, p < 0.01, power = 0.99, Table 2). In the cohort with viral etiology of HCC, CMKLR1 protein tended to decline in HCC tissues of HBV, but not of HCV, infected patients (Figure 6D,E and Table 2). 

A strong downregulation of CMKLR1 protein was noticed in tissues of patients infected with both viruses (Table 2). Interestingly, CMKLR1 protein was low in tumor and non-tumor tissues of HCV infected patients in comparison to the respective tissues of HBV and HBV/HCV infected patients (Figure 6D). Of note, CMKLR1 protein in the tumors did not correlate with TNM classification in the non-virus and the virus-related HCC cohorts (Figure 6F,G), and when associations were analyzed separately for HBV infected patients (Figure 6H).

### 2.7. Effect of Tazarotene and Retinoic Acid on Hepatocyte Chemerin and CMKLR1 Protein

**Figure 7:**
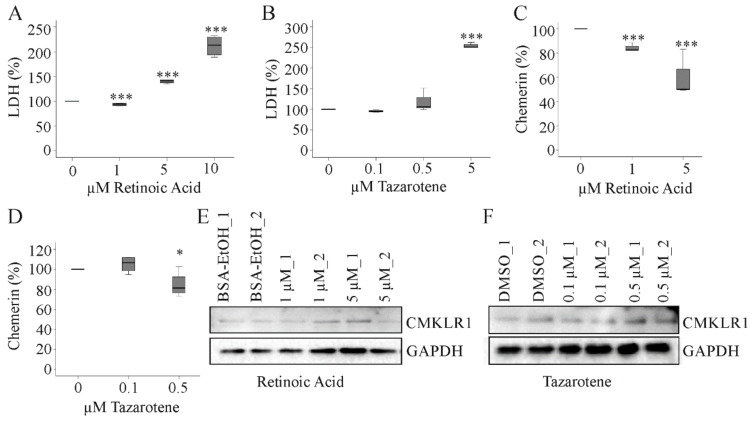
Effect of retinoic acid and tazarotene on chemerin and CMKLR1. (**a**) HepG2 cells were incubated with increasing concentrations of retinoic acid for 24 h and LDH was measured in the supernatants (n = 4); (**b**) HepG2 cells were incubated with increasing concentrations of tazarotene for 24 h and LDH was measured in the supernatants (n = 4); (**c**) HepG2 cells were incubated with retinoic acid (1 and 5 µM) for 24 h and chemerin was measured in the supernatants (n = 4); (**d**) HepG2 cells were incubated with tazarotene (0.1 and 0.5 µM) for 24 h and chemerin was measured in the supernatants (n = 4); (**e**) HepG2 cells were incubated with retinoic acid (1 and 5 µM) for 24 h and CMKLR1 was analyzed by immunoblot (n = 2); (**f**) HepG2 cells were incubated with tazarotene (0.1 and 0.5 µM) for 24 h and CMKLR1 was analyzed by immunoblot (n = 2). Bovine serum albumin (BSA) dissolved in EtOH and dimethylsulfoxid (DMSO) served as solvent controls. * p < 0.05, *** p < 0.001. Statistical test used: Paired t-test.

Chemerin is a tazarotene-induced protein in the skin. Tazarotenes are synthetic retinoids and chemerin was identified as a retinoic acid receptor responder protein [25]. Retinoids protect from HCC and proteins regulated by natural or synthetic retinoids may mediate the antitumor effects of retinoids [26,27]. Whether tazarotene or retinoic acid induce chemerin in hepatocytes was not tested as far as we know. Retinoic acid increased soluble lactate dehydrogenase (LDH) in HepG2 cells at a concentration of 5 and 10 µM. At 1 µM there was a modest, but highly significant decline of LDH (Figure 7A). Retinoids are important for cell growth and low levels seem to exert beneficial effects in HepG2 cells [27]. Tazarotene led to elevation of LDH levels at a concentration of 5 µM whereas 0.1 and 0.5 µM had no effect on cell viability (Figure 7B). Soluble chemerin declined in HepG2 cells treated with 1 or 5 µM retinoic acid or 0.5 µM tazarotene (Figure 7C,D). Retinoic acid and tazarotene did not affect cellular CMKLR1 protein in HepG2 cells (Figure 7E,F).

## 3. Discussion

The present analysis found higher chemerin protein in primary liver tumors of patients with cryptic, NAFLD and HBV related etiology. In HCV-associated HCC chemerin levels were essentially unaltered. Moreover, CMKLR1 protein was lower in the tumors than the adjacent non-tumor tissues of non-viral HCC patients, and tended to decrease in HBV-related tumors. Such a regulation did not exist in HCV patients. The power value for chemerin upregulation in patients with unknown etiology was 0.5. The desired value is 0.8 [28] and further analysis is required to prove whether chemerin protein is indeed induced in the tumors.

The present findings are contrary to earlier studies describing a decline of chemerin in HCC tissues of the majority of patients [8,9,12]. In opposition to expectation we did not identify lower chemerin in any of the tumors.

A possible explanation could be provided as antibodies with different specificities were used in the previous studies [8,9,12]. The chemerin antibody applied in the current investigations detected different chemerin isoforms with a high sensitivity and specificity. This antibody was also employed in a recent study, that reported decreased chemerin in HCC tissues [9]. Moreover, the different antibodies used in this study consistently revealed an upregulation of chemerin in the tumor tissues.

Chemerin protein in hepatic tumors tended to be higher in a murine model of diethylnitrosamine-induced liver cancer [7]. In a NASH-HCC model, tumor and paratumor chemerin levels were basically the same [21]. One possible explanation is that chemerin regulation in murine liver cancer tissues is related to disease etiology. It can, however, not be ruled out that chemerin expression in HCC tissues was associated with tumor size in rodents. The tumors were larger in the non-NASH HCC model because the experiments were terminated four months later [7,21]. Chemerin in murine HCC tissues was not significantly changed, and these animal models are not appropriate to further study the expression of chemerin in liver tumors.

Remarkably, upregulation of chemerin in the tumors was highest in patients with NAFLD. An association of chemerin and liver steatosis was not observed in virus infected patients. This implies that chemerin may be particularly important in NAFLD related HCC. Though the number of patients was rather small, the power value of this finding was above 0.95 suggesting a high probability that chemerin is induced in NAFLD-HCC. However, there was a strong decline of CMKLR1 protein in these tumors, and this may indicate impaired chemerin signalling in HCC tissues.

Chemerin is produced as an inactive protein and needs to be cleaved at its C-terminus to become active. Proteolysis generates different chemerin variants [1]. To ensure that different chemerin isoforms were detected by the three antibodies, full-length inactive chemerin (chemerin-163), the active chemerin isoforms (chemerin-157 and -156) and a shorter, inactive variant (chemerin-155) were expressed in HepG2 cells. The polyclonal goat antibody detected all variants with a high specificity and sensitivity. The signal-to-noise ratio was suboptimal for the two other antibodies tested. The polyclonal antibody from Abcam mainly bound to chemerin-163. The monoclonal antibody from R&D Systems did not convincingly detect any of the recombinant isoforms. Different immunoblot protocols may improve the experimental quality of these two antibodies to detect chemerin variants, but this was not examined in the present study.

Chemerin isoform distribution was described in human serum and adipose tissues, but not in the liver [11]. Moreover, chemerin processing may differ in tumor and para-tumor tissues. Until now there is no proof that hepatic chemerin is biologically active and whether higher levels in the tumors are indeed associated with increased activity. In murine HCC tissues chemerin-156 was not detected and chemerin-155 was the abundant active isoform [7]. Whether this applies to human tissues was not analyzed so far. Of note, CMKLR1 was suppressed in HCC tissues of most patients and chemerin / GPR1 may be the predominant signaling route in liver tumors. Interestingly, CMKLR1 was also low in advanced murine HCCs [7].

CMKLR1 protein was reduced in tumor and non-tumor liver tissue of HCV patients in comparison to HBV and double infected patients. Kukla et al. described that CMKLR1 mRNA was expressed in the liver of HCV infected patients [29]. CMKLR1 mRNA expression was not associated with necroinflammatory activity, steatosis grade and fibrosis stage [29]. Moreover, it was not possible to define the liver cells with low CMKLR1 levels upon HCV infection because whole liver tissues were used for the study. CMKLR1 is expressed by hepatocytes, Kupffer cells, endothelial cells and hepatic stellate cells [30]. Flow cytometry analysis or immunohistology of CMKLR1 can identify the specific cell type with low CMKLR1 expression in HCV liver.

Discordant results regarding chemerin levels in primary liver tumors may need a closer examination of the patients. An obvious difference between the present cohort and the patients enrolled in previous studies is ancestry [8,9,12]. Asian patients have a higher incidence of HCC [31]. Moreover, tumor mutations differed in Asian and European patients [31]. Several genes were exclusively mutated in Asians, and these patients had a higher frequency of TP53 and RB1 mutations [31]. Whether these differences account for the opposite regulation of chemerin in HCC is completely unexplored so far. Interestingly, HCC etiology had no impact on the ethnic mutational differences [31] and thus may not contribute to hepatic expression of chemerin in liver tumors.

Aberrant promoter methylation is a hallmark of cancer, and was demonstrated to control constitutive chemerin expression in hepatocytes [32]. Maternal smoking during pregnancy was associated with reduced chemerin promoter methylation in the skin and induced gene expression [33]. Thus, comparison of chemerin promoter methylation in tumors and adjacent tissues of patients with Asian and European ancestry may resolve why chemerin is differentially regulated in HCC.

Tumor chemerin of the present cohort was not related to TNM stage indicating that chemerin might not be relevant for tumor progression. Though low tumor chemerin was correlated with larger tumor size, less differentiated tumors and shorter survival in a recent study [12], there were no associations of tumor chemerin and TNM stage [12]. At present, the factors affecting chemerin regulation in human HCC are unknown. Disease etiology, medication, progression of HCC, smoking and current or previous therapies may have to be considered to further clarify this issue.

Fatty acid loading, leptin, TNF and TGF beta did not regulate chemerin protein in primary human hepatocytes [34]. Leptin, IL-6, TNF and TGF beta could not change cellular chemerin protein in HepG2 cells [20]. Thus, higher chemerin in HCC tissues is neither induced by the inflammatory tumor microenvironment nor by fibrotic molecules.

Of note, retinoic acid and tazarotene exert anti-tumor functions and induced chemerin in keratinocytes [35,36]. These agents reduced the viability of HepG2 cells without enhancing chemerin production. Indeed, soluble chemerin protein even declined. Recent experiments showed that active chemerin did not affect Hepa1-6, HepG2 and Huh7 cell proliferation and apoptosis [9,23]. Hence, chemerin has no direct effect on the viability of tumor cells.

Biologic active recombinant murine chemerin-156 (the murine homolog of human chemerin-157) exerted anti-tumor effects in experimental mouse models [7,8,9]. Up to now there is no proof that hepatic chemerin is active in humans. Present results report higher chemerin in HCC tissues of European patients with NAFLD and HBV etiology but not in HCV related HCC. Future studies are required to analyse chemerin activity and isoform distribution in human HCCs of different etiologies.

## 4. Materials and Methods

### 4.1. Human HCC Tissues

Human HCC tissues and para-tumorous tissues of patients infected with HBV (10 patients), HCV (11 patients), HB/CV (three patients) and non-infected patients (21 patients), which were all treated at the University Hospital of Regensburg, were analyzed in the present study. Age did not differ between the three groups. BMI had a trend to be higher in non-viral than viral HCC. In viral HCC, tissues of male and female patients were obtained whereas only males were enrolled in the non-viral patient cohort. HCC is more common in males [13] and gender did not differ between the cohorts. Fibrosis stage was higher in HCV than HBV and non-viral HCC patients. Grading and TNM stage were similar in all groups. Steatosis grade was comparable in patients with viral infections, and was higher in non-viral than HBV-HCC patients. The characteristics of the cohorts are given in Table 1. The non-viral HCC cohort was already described, and in this previous study lipid species were analyzed in the liver tissues [37]. TNM classification was done as described [38]. Experimental procedures were performed according to the guidelines of the charitable state controlled foundation Human Tissue and Cell Research (HTCR), with the written informed patient’s consent. The study was approved by the local ethical committee (University Hospital of Regensburg, Ethic code: 15-101-0052).

### 4.2. Cell Lines and Primary Human Hepatocytes

HepG2, Huh7, Hep3B and PLC/PRF/5 cells were from the American Type Culture Collection (ATCC, Middlesex, UK). PLC/PRF/5 cells were cultivated in RPMI 1640 medium supplemented with 10% fetal bovine serum, penicillin (200 U/mL medium) and streptomycin (0.2 mg/mL medium). HepG2, Huh7 and Hep3B cells were cultivated in DMEM medium supplemented with 10% fetal bovine serum and 1% penicillin/streptomycin. Primary human hepatocytes were cultivated as described in detail [39,40].

### 4.3. Transfection of Cells with siRNA

Primary human hepatocytes were transfected by electroporation as described [41]. Cell lines were transfected by Lipofectamine^TM^ 3000 Reagent (Thermo Fisher Scientific, Schwerte, Germany) as suggested by the supplier. Chemerin siRNA (GAAGAAACCCGAGUGCAAAtt) was ordered from Thermo Fisher Scientific.

### 4.4. Recombinant Expression of Chemerin Isoforms in HepG2 Cells

Polymerase chain reaction to amplify chemerin cDNA was done with the universe primer 5’-CGA AAG CTT ATG CGA CGG CTG CTG ATC C-3’ and the reverse primers chemerin-163:5’-CGA CCG CGG TTA GCT GCG GGG CAG G-3’, chemerin-157:5’-CGA CCG CGG TTA GGA GAA GGC GAA CTG TCC AGG-3’, chemerin-156:5’-CGA CCG CGG TTA GAA GGC GAA CTG TCC AGG GAA-3’ and chemerin-155:5’-CGA CCG CGG TTA GGC GAA CTG TCC AGG GAA GTA-3’. DNA was cloned in the plasmid pcDNA3.1 (Thermo Fisher Scientific). The cutting sites for the restriction enzymes are underlined and fragments were cloned with HindIII and SacII. The DNA sequences of the cloned fragments were verified by sequencing (Thermo Fisher Scientific, Regensburg, Germany). Expression of chemerin variants in HepG2 cells was described recently [23].

### 4.5. Tazarotene and Retinoic Acid

Retinoic acid and the synthetic retinoid tazarotene were ordered from Tocris Bioscience (Wiesbaden-Nordenstadt, Germany).

### 4.6. SDS-PAGE and Immunoblotting

SDS-polyacrylamide gel electrophoresis (SDS-PAGE), transfer to PVDF membranes (Bio-Rad, Munich, Germany) and incubations with antibodies were performed as described [37]. Detection of the immune complexes employed the ECL western blot detection system (Amersham Pharmacia, Deisenhofen, Germany). Quantification of signals was done using ImageJ software [42]. A polyclonal (AF2324, RRID: AB_416577) and a monoclonal chemerin antibody (MAB2324, RRID: AB_2175697) were from R&D Systems (Wiesbaden, Germany). A polyclonal chemerin antibody was also ordered from Abcam (ab72965; Cambridge, UK, RRID:AB_1523346). GAPDH, β-actin, CMKLR1 and cyclophilin A antibodies were from New England Biolabs GmbH (Frankfurt, Germany). The original Western blot figures can be found in the Supplementary Materials.

### 4.7. ELISA

Chemerin ELISA was ordered from R&D Systems and was performed as recommended by the company. Serum was diluted 1:250-fold and all samples were measured in duplicate.

### 4.8. Real-Time RT-PCR.

Real-time RT-PCR was performed as described [20,43]. Serial dilutions of liver cDNA were used for quantification. This allows to correct for different efficiencies of independent assays. For normalization, tyrosine 3-monooxygenase/tryptophan 5-monooxygenase activation protein, zeta polypeptide (YWHAZ) mRNA was used and was amplified with 5’-GCA ATT ACT GAG AGA CAA CTT GAC A-3′ and 5´-TGG AAG GCC GGT TAA TTT T-3’.

### 4.9. Statistical Analysis

Data are presented as box plots. Box plots display the median values, lower and upper quartiles and the range of the values. Statistical analysis used Mann-Whitney U Test, one-way ANOVA with posthoc Bonferroni test (SPSS Statistics 25.0 program, IBM, Leibniz Rechenzentrum, München, Germany) or Student’s t-test (MS Excel), and a value of *p* < 0.05 was regarded as significant. Chi-square test was applied to test for the relationships between categorical variables. To calculate power values Wilcoxon singed-rank test for matched pairs (G*Power 3.1.6, [44]) was used.

## 5. Conclusions

Chemerin protein is induced in the tumors of HBV and NAFLD related European HCC patients. This is accompanied by suppression of CMKLR1 protein in HCC tissues of NAFLD patients. In HCV-HCC chemerin is not regulated and CMKLR1 is already low in the non-tumor tissues of those patients. Regarding the anti-tumor effects of chemerin in experimental HCC models, drugs to enhance CMKLR1 abundance may reduce disease severity.

## Figures and Tables

**Table 1:** Characteristics of the study populations. Median values and range are given. When data of the whole group were not documented, the respective number of patients where these data were available is shown in superscript. Abbreviations used: Alanine aminotransferase, ALT; aspartate aminotransferase, AST; body mass index, BMI; Hepatitis B virus, HBV; Hepatitis C virus, HCV; not documented, nd * Significance: HBV compared to HCV: *p < 0.05, ** *p* < 0.01. HBV compared to HB/CV: %%% *p* < 0.001. HBV compared to non-viral $ *p* < 0.05, HCV compared to non-viral: && *p* < 0.01, &&& *p* < 0.001. Statistical test used: One-way ANOVA with posthoc Bonferroni.

Patients’ Characteristics	HBV	HCV	HBV/HCV	Non-Viral
Number	10	11	3	21
Sex (male/female)	8/2	8/3	2/1	21/0
Type 2 diabetes	2	3	0	11
Age (years)	60 (35–78)	54 (48–71)	61 (54–76)	63 (47–84)
BMI (kg/m²)	24.5 (18.7–29.4)	25.2 (18.8–28.7)	26.2 (22.4–30.0)	27.7 (19.7–44.6)
AST (U/l)	40 (19–103)**^, %%%^	75 (39–151)**^, &&^	95 (78–200)^%%%^	31.5 (14–145)^20^^&&^
ALT (U/l)	40 (24–23)^%^	60 (27–145)	134 (66–167)^%^	42 (23–378)^20^
Bilirubin (mg/dl)	0.7 (0.3–1.6)	0.7 (0.4–3.7)	0.5 (0.5–0.8)	0.6 (0.2–2.5)^20^
Steatosis Grade 0/1/2/3/nd	7/1/2/0^$^	6/2/1/1/1	2/0/0/1/0	11/3/3/4^$^
Fibrosis Stage 0/1/2/3/4	2/2/0/3/3*^,$^	0/0/0/3/8*^,&&&^	0/0/1/1/1	8/10/1/2^$, &&&^
Grading G1/G2/nd	1/4/5	0/8/3	0/1/2	3/14/4
TNM Stage I/II/IIIa/ nd	3/6/1/0	4/3/4/0	1/0/1/1	9/6/5/1

**Table 2:** Post hoc Power Analysis using G*Power 3.1.6. T Test-means: Wilcoxon signed-rank test (matched pairs). Output parameters of the analysis are shown for the different groups.

Output of Power Calculation	Cryptic	NAFLD	HBV	HCV	HBV/HCV
**Chemerin**					
Noncentrality parameter δ	2.22	4.61	3.55	1.44	1.20
Critical t	2.24	2.28	2.28	2.24	4.62
Df	9.50	8.54	8.55	9.50	1.89
Power (1-β err prob)	0.51	0.98	0.88	0.25	0.11
CMKLR1					
Noncentrality parameter δ	4.25	6.33	1.11	0.60	3.97
Critical t	2.28	2.24	2.28	2.24	4.62
Df	8.55	9.50	8.55	9.50	1.86
Power (1-β err prob)	0.96	0.99	0.17	0.08	0.52

## Supplementary Materials

The following are available online at https://www.mdpi.com/2072-6694/12/10/2967/s1, Supplementary file: The original Western blot figures.
