# Supplementary figures and images for: Chemerin Is Induced in Non-Alcoholic Fatty Liver Disease and Hepatitis B-Related Hepatocellular Carcinoma
Source: Cancers (Basel). 2020 Oct 13;12(10):2967. doi: 10.3390/cancers12102967 (PMC7602083; doi:10.3390/cancers12102967)

Figure 1B

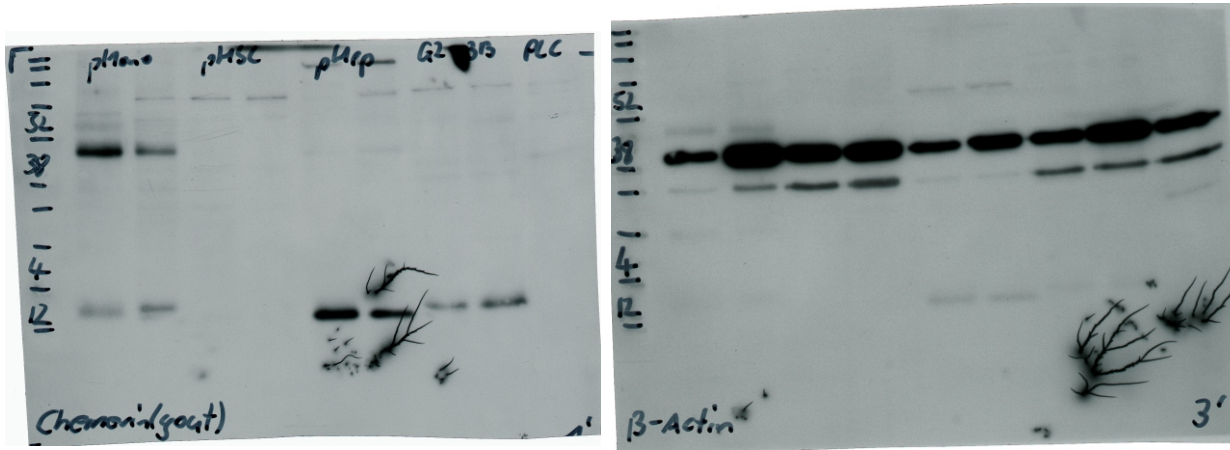

Figure 1C

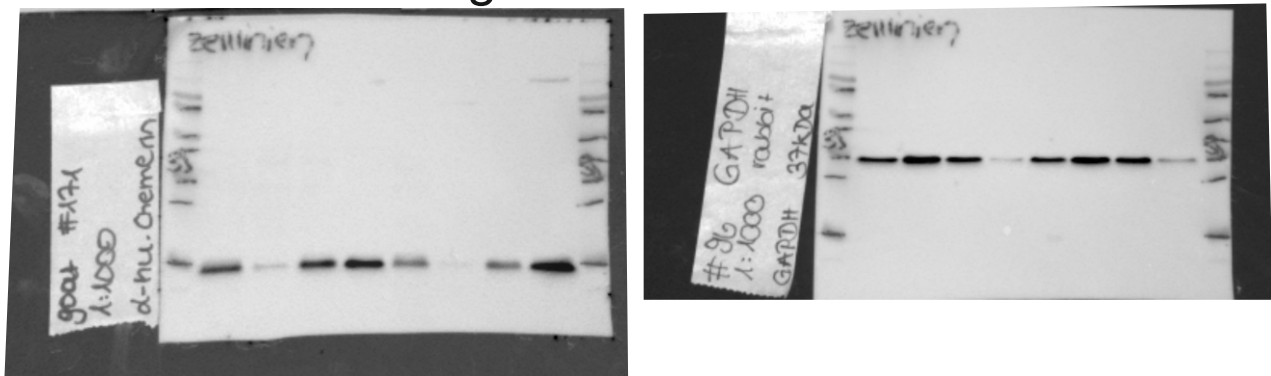

Figure 1E

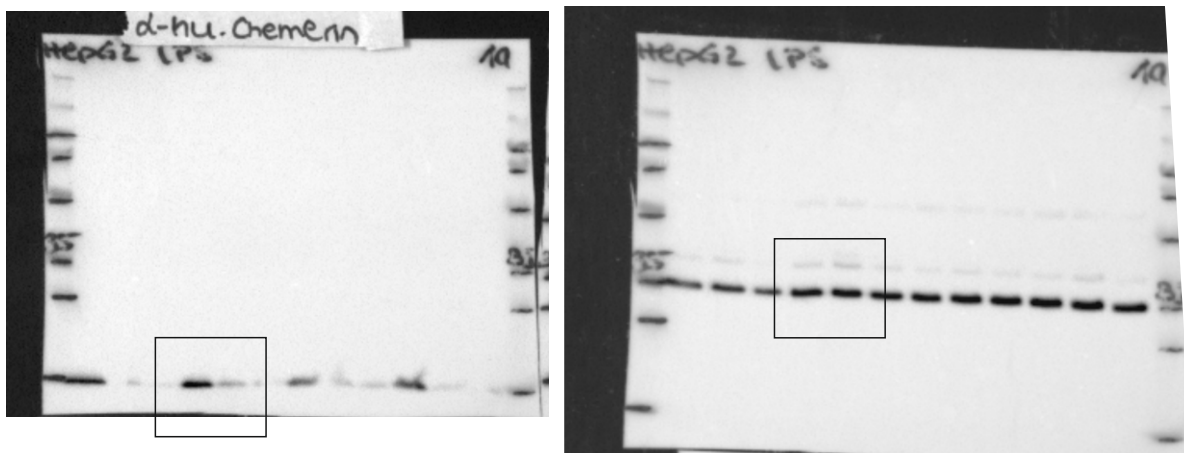

Figure 1F

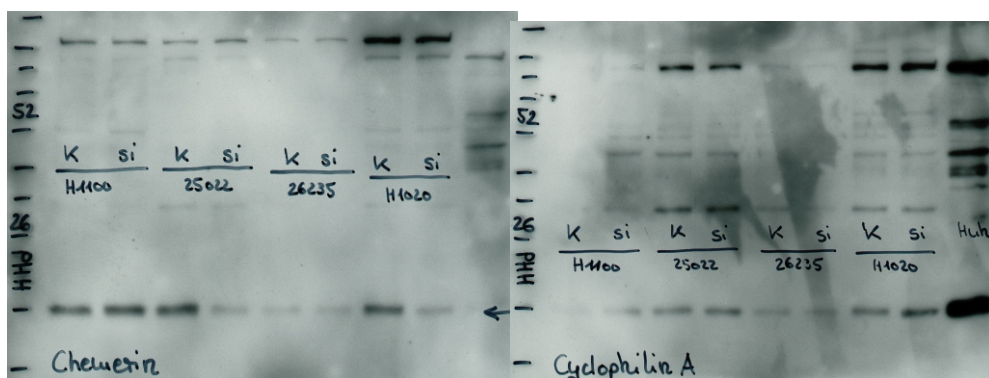

Figure 1G

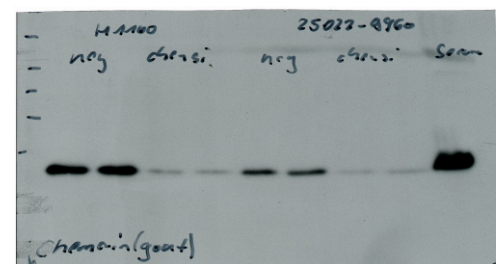

# Figure 2B

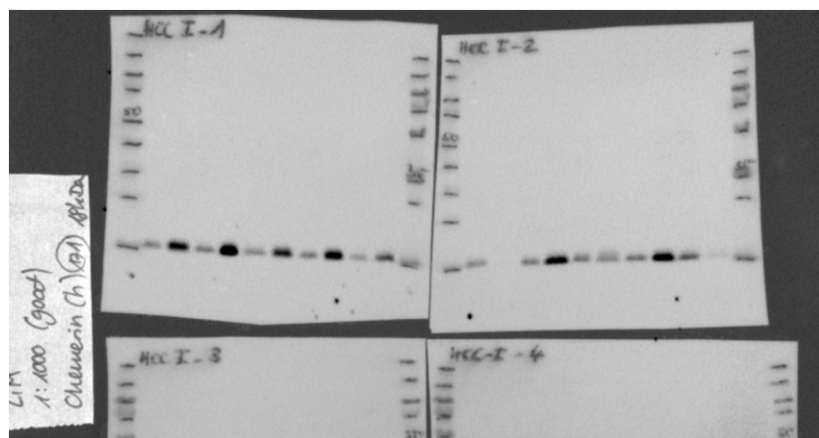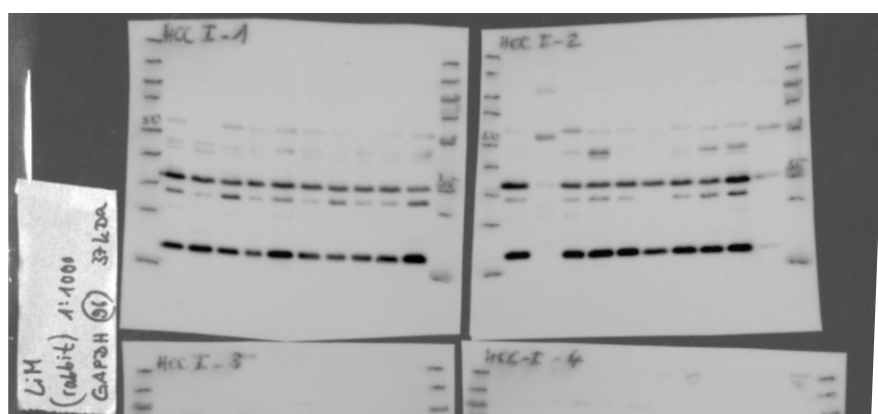

# Figure 3A

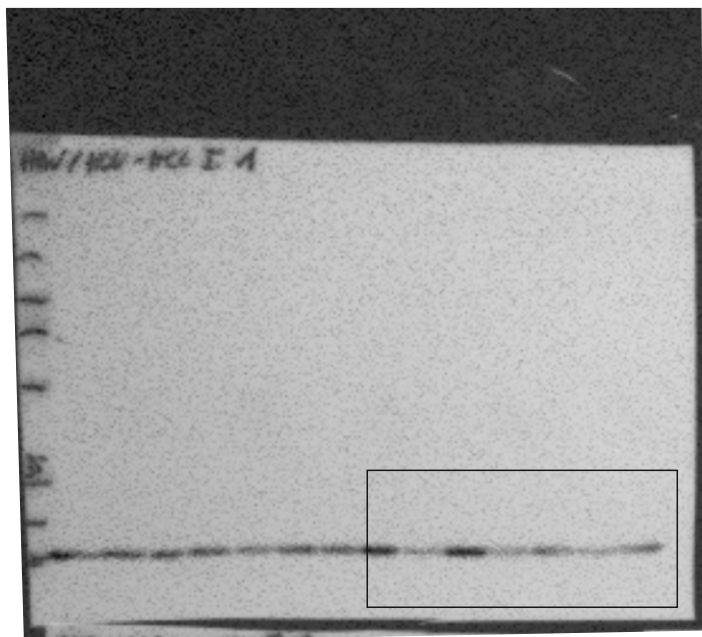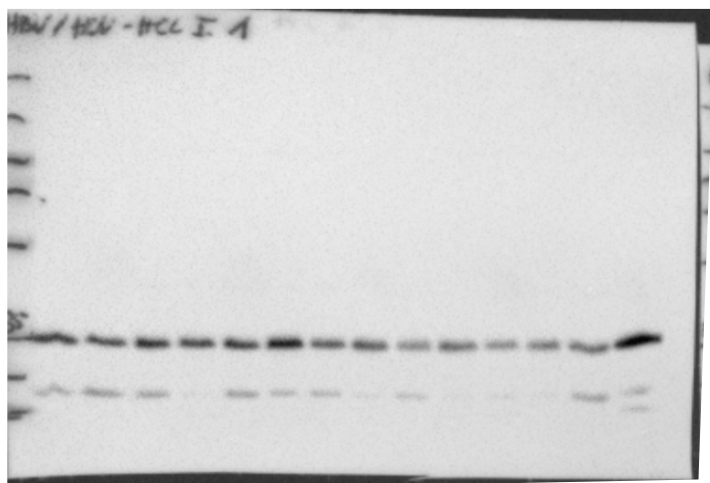

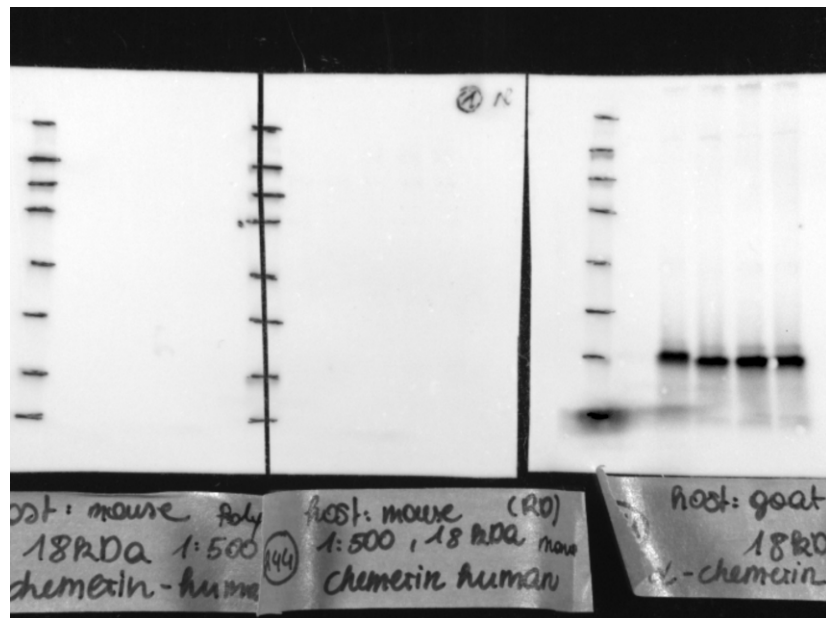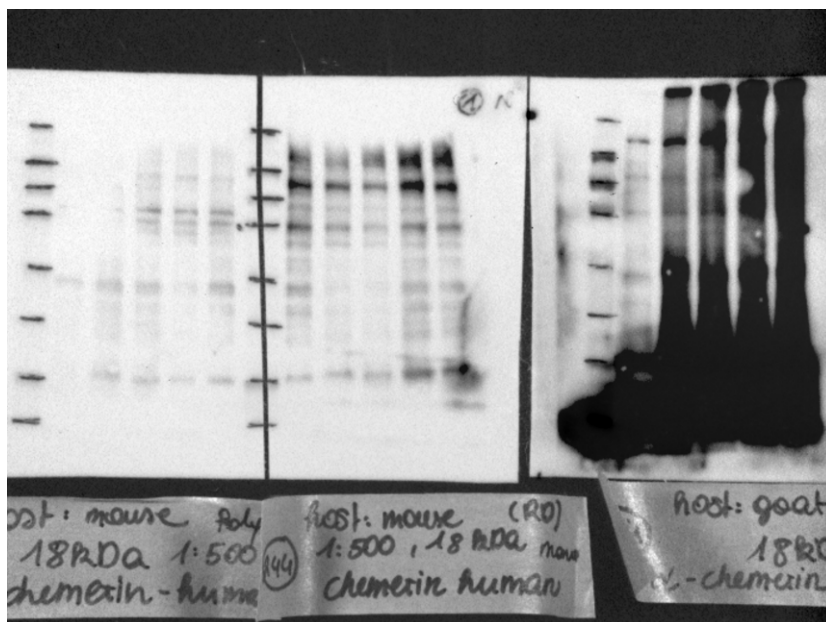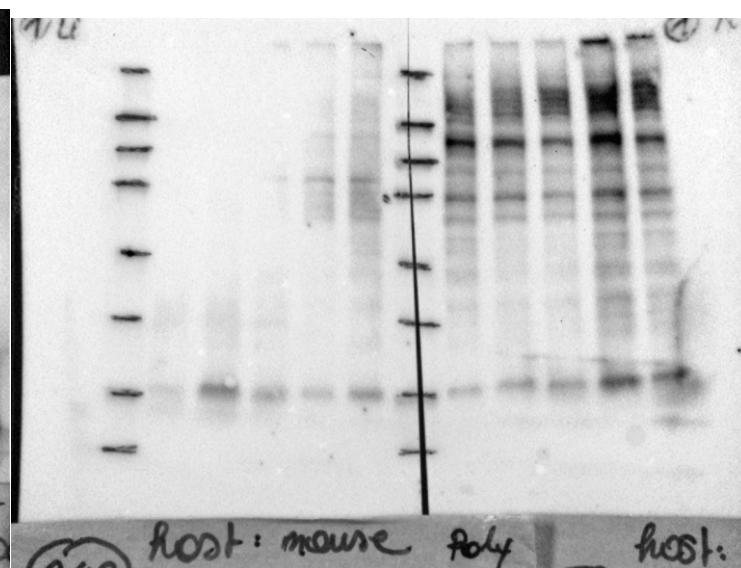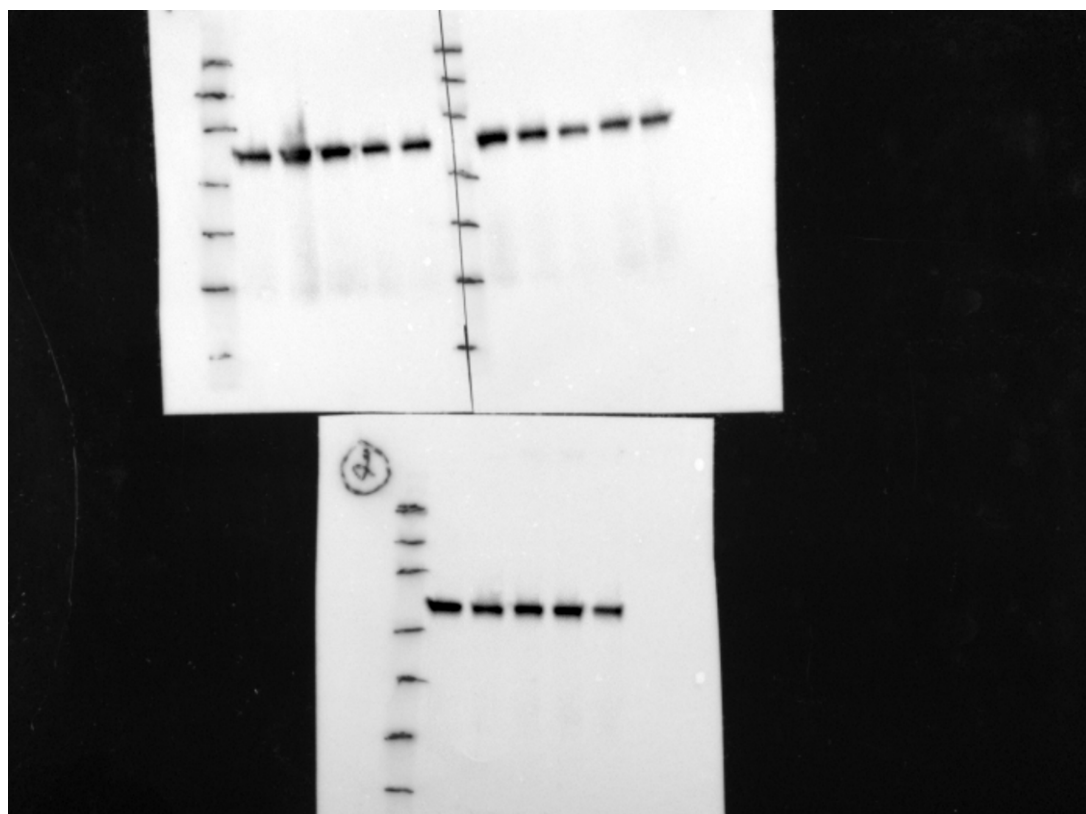

Figure 5

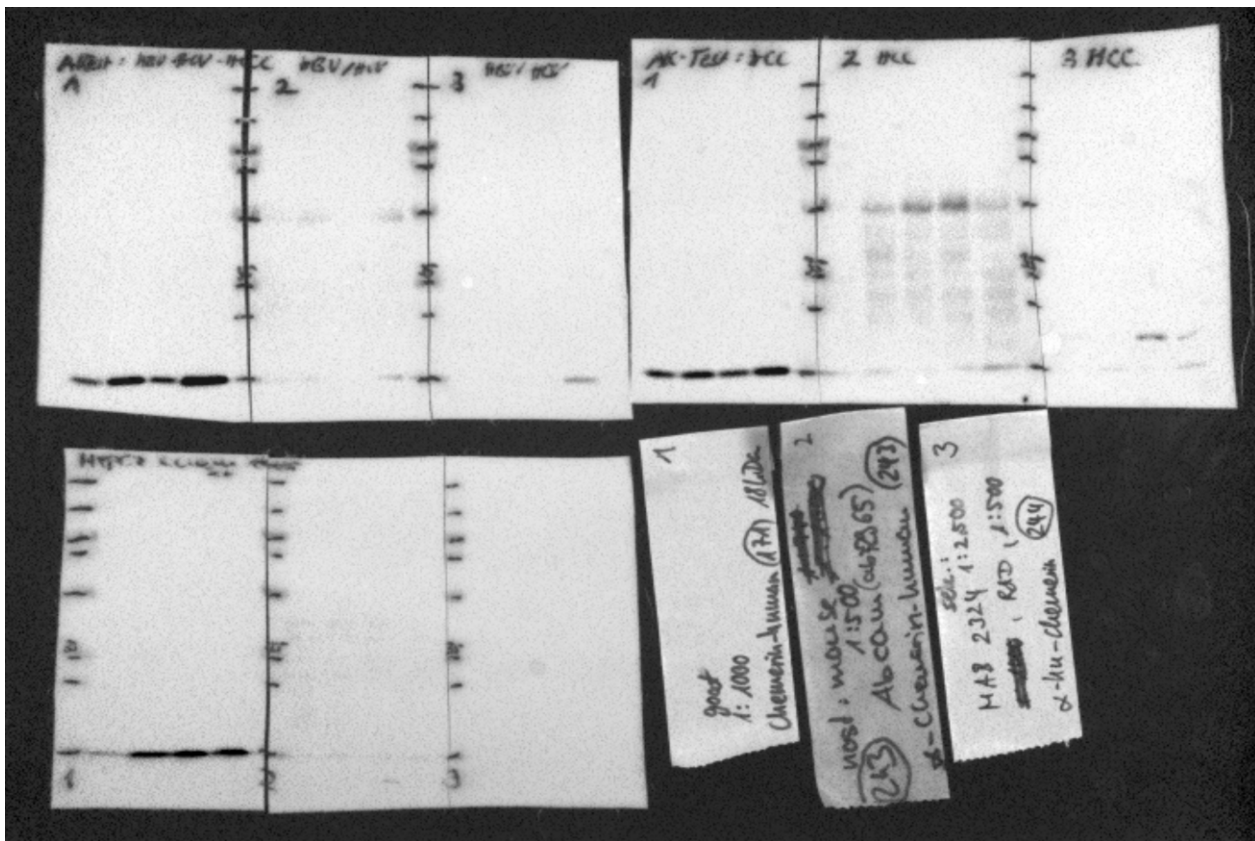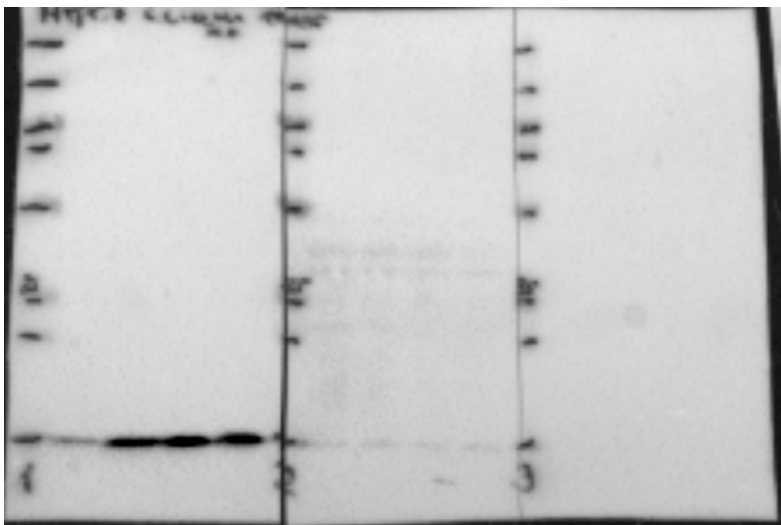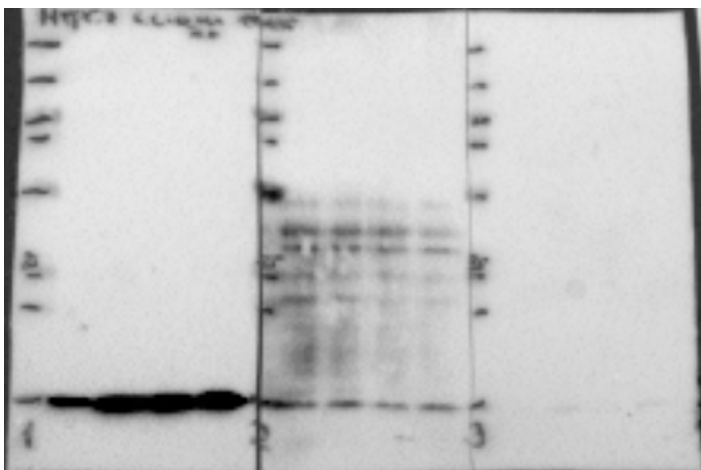

Figure 6C

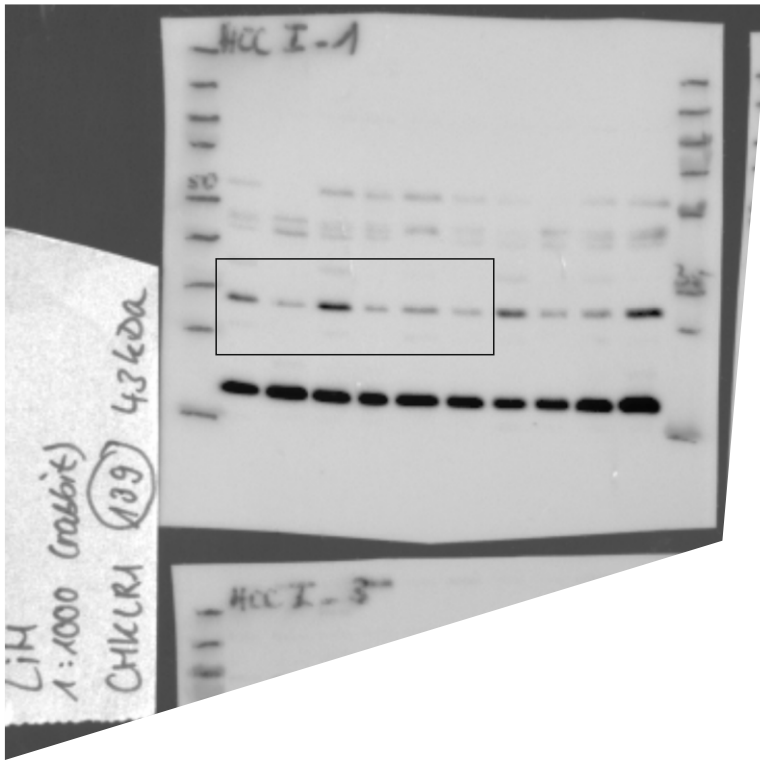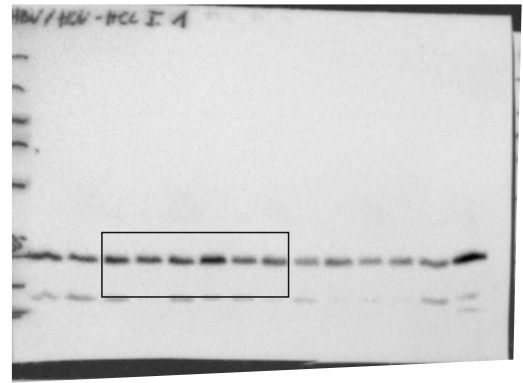

Figure 6A

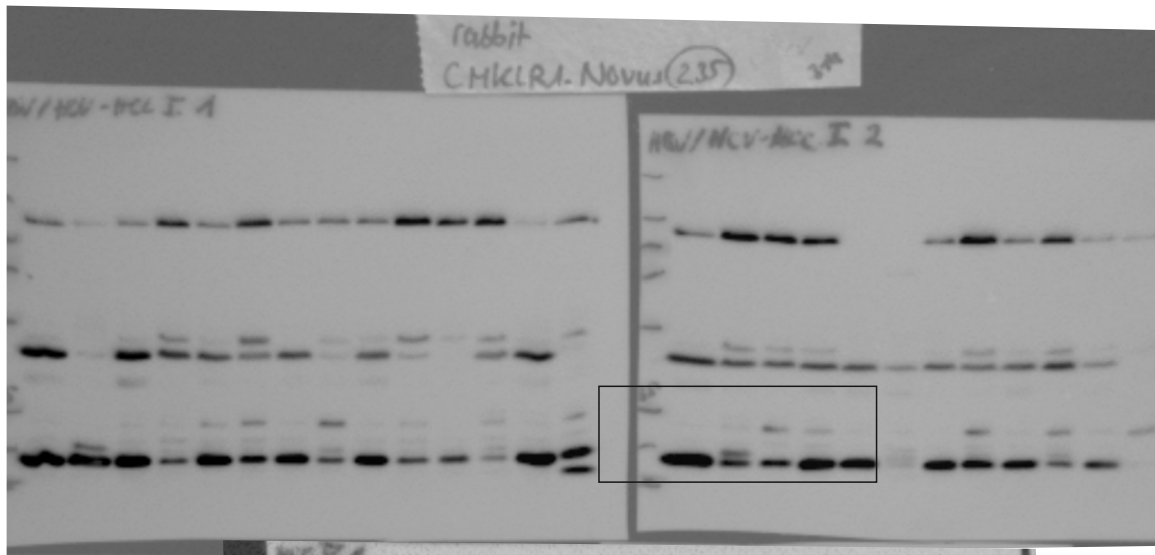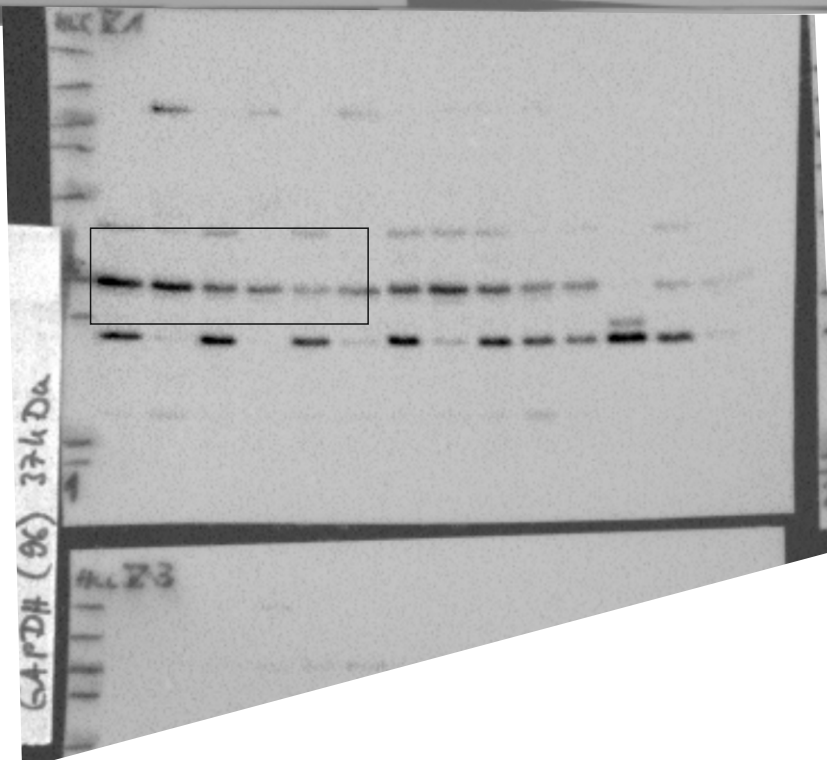

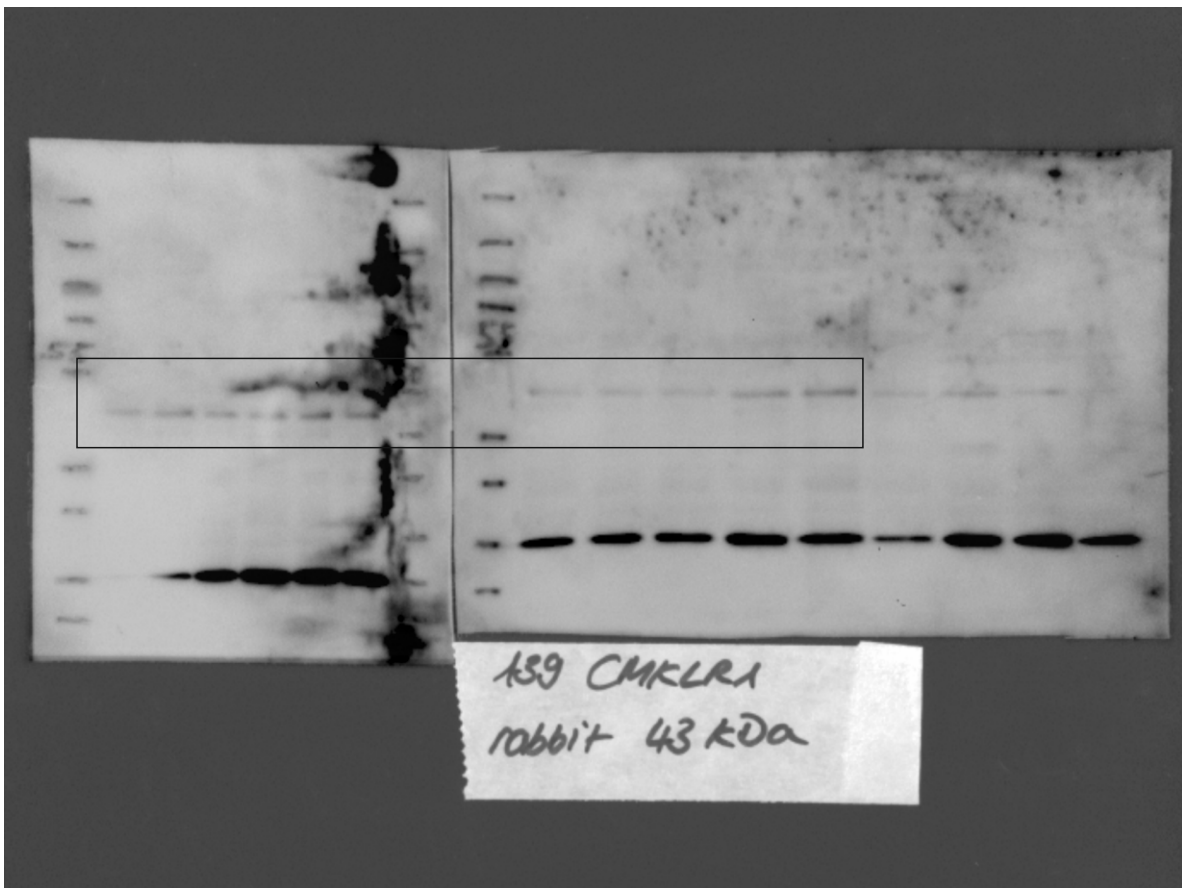

Figure 7

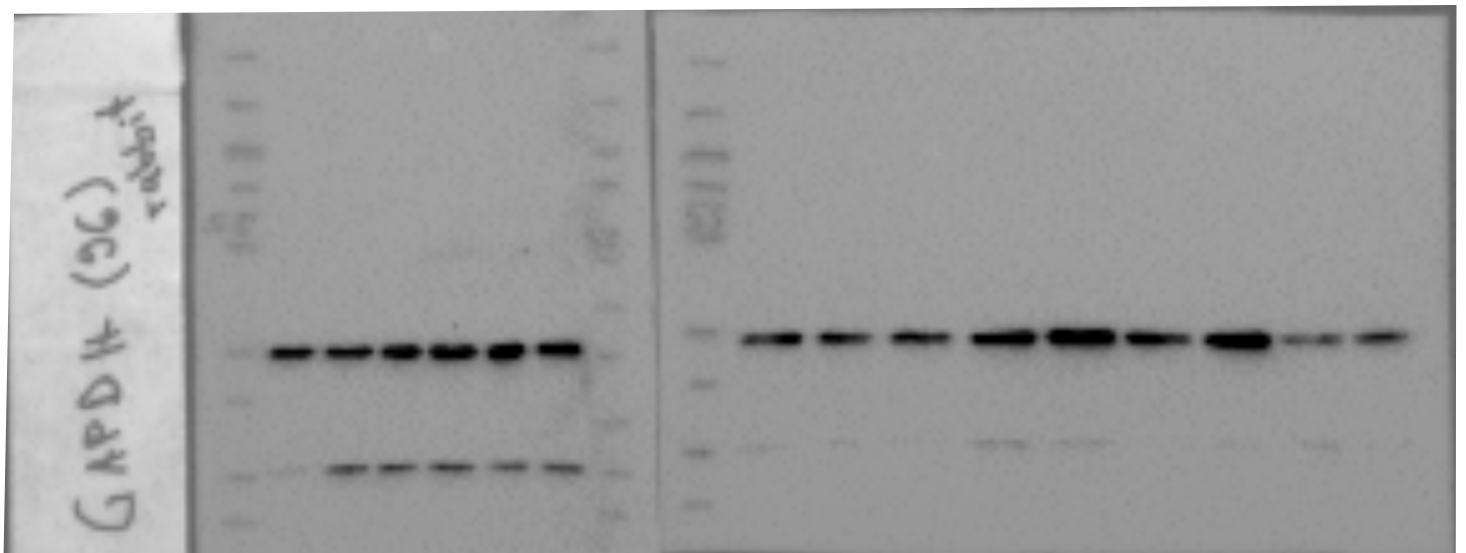

Supplement: Supplementary file 1 [file cancers-12-02967-s001.pdf]
